# Dietary Saturated Fats and Health: Are the U.S. Guidelines Evidence-Based?

**DOI:** 10.3390/nu13103305

**Published:** 2021-09-22

**Authors:** Arne Astrup, Nina Teicholz, Faidon Magkos, Dennis M. Bier, J. Thomas Brenna, Janet C. King, Andrew Mente, José M. Ordovas, Jeff S. Volek, Salim Yusuf, Ronald M. Krauss

**Affiliations:** 1 Healthy Weight Center, Novo Nordisk Foundation, Tuborg Havnevej 19, 2900 Hellerup, Denmark; 2 The Nutrition Coalition, New York, NY 10011, USA; nina@nutritioncoalition.us; 3 Department of Nutrition, Exercise and Sports, University of Copenhagen, 1958 Frederiksberg C, Denmark; fma@nexs.ku.dk; 4 Children’s Nutrition Research Center, Department of Pediatrics, Baylor College of Medicine, Houston, TX 77030, USA; dbier@bcm.edu; 5 Department of Pediatrics, Dell Pediatric Research Institute, University of Texas at Austin, Austin, TX 78723, USA; tbrenna@utexas.edu; 6 Department of Chemistry, Dell Pediatric Research Institute, University of Texas at Austin, Austin, TX 78723, USA; 7 Department of Nutrition, Dell Pediatric Research Institute, University of Texas at Austin, Austin, TX 78723, USA; 8 Department of Nutritional Sciences and Toxicology, University of California-Berkeley, Berkeley, CA 94720, USA; jking829@berkeley.edu; 9 Population Health Research Institute, Hamilton Health Sciences, Hamilton, ON L8L 2X2, Canada; Andrew.Mente@phri.ca (A.M.); Salim.Yusuf@phri.ca (S.Y.); 10 Department of Health Research Methods, Evidence, and Impact, McMaster University, Hamilton, ON L8S 4L8, Canada; 11 Nutrition and Genomics Laboratory, Human Nutrition Research Center of Aging, Tufts University, Boston, MA 02111, USA; Jose.Ordovas@tufts.edu; 12 IMDEA Food Institute, 28049 Madrid, Spain; 13 Department of Human Sciences, Ohio State University, Columbus, OH 43210, USA; volek.1@osu.edu; 14 Department of Medicine, McMaster University, Hamilton, ON L8S 4L8, Canada; 15 Department of Pediatrics, University of California-San Francisco, San Francisco, CA 94609, USA; ronald.krauss@ucsf.edu; 16 Department of Medicine, University of California-San Francisco, San Francisco, CA 94609, USA

**Keywords:** saturated fats, polyunsaturated fats, dietary guidelines, Dietary Guidelines for Americans, nutrition guidelines, cardiovascular disease, heart disease, evidence-based

## Abstract

The last decade has seen nearly 20 papers reviewing the totality of the data on saturated fats and cardiovascular outcomes, which, altogether, have demonstrated a lack of rigorous evidence to support continued recommendations either to limit the consumption of saturated fatty acids or to replace them with polyunsaturated fatty acids. These papers were unfortunately not considered by the process leading to the most recent U.S. Dietary Guidelines for Americans, the country’s national nutrition policy, which recently reconfirmed its recommendation to limit saturated fats to 10% or less of total energy intake, based on insufficient and inconsistent evidence. Continuation of a cap on saturated fat intake also fails to consider the important effects of the food matrix and the overall dietary pattern in which saturated fatty acids are consumed.

## 1. Introduction

Since the introduction of the U.S. Dietary Guidelines for Americans (DGA) in 1980, national nutrition policy has consistently advised limiting saturated fat consumption as a central strategy for reducing risk for atherosclerotic cardiovascular disease (CVD). Saturated fatty acids are defined as those molecules that are “saturated” by hydrogen, without any double bonds. They are found in all foods but are especially concentrated in dairy, red meat, and the so-called tropical oils. In 1990, the DGA added a specific cap limiting these fats to 10% of calories [1] which has remained in place ever since, including in the recent 9th edition of the DGA released in late December 2020 by the U.S. Departments of Agriculture and Health and Human Services (USDA-HHS) [2]. The guidelines are updated every five years, and the policy must, according to U.S. law, serve the “general public” and reflect “the preponderance of the scientific and medical knowledge which is current at the time the report is prepared” [3]. This review aims to address the current knowledge regarding the effects of dietary saturated fats on heart disease as well as the consideration of these data by recent Dietary Guidelines Advisory Committees (DGACs).

## 2. Historical Perspective on Dietary Saturated Fat Recommendations

The hypothesis that saturated fats cause CVD emerged in the late 1950s, when scientists observed that these fats tend to raise the concentration of total serum cholesterol, which in turn was considered a potent risk factor for heart disease. Ancel Keys, a physiologist at the University of Minnesota, postulated that saturated fats along with dietary cholesterol were the principal causes of cardiovascular disease, and his “diet-heart hypothesis” was adopted by leading groups, including the American Heart Association. At the time, the evidence supporting this advice consisted primarily of one observational study which compared the level of saturated fats intake with heart-disease outcomes in seven countries and involved 12,763 men in Europe, the U.S. and Japan [4] (pp. 41–42). This study recorded dietary data in less than 5% of the participants, corresponding to a total of about 500 individuals, or fewer than 100 participants per country. The study claimed an association between saturated fat consumption and heart disease, yet because the Seven Countries Study (SCS) was not a clinical trial, it could not demonstrate cause-and-effect. Still, the study was enormously influential in the field of nutrition for its groundbreaking, far-ranging work, even though numerous methodological shortcomings were later identified, including the non-random selection of countries for the study, the inclusion of only men, the collection of dietary data from less than 5% of the total sample, the use of non-standardized and non-validated methods for collecting dietary data, the lack of contemporary statistical approaches to minimize confounding, and inconsistent methods of follow-up [5]. The results of the SCS have never been independently analyzed, and there are several more recent studies using similar approaches that have failed to confirm its conclusions, as described below [6].

Recognizing the need for more rigorous, clinical-trial data, governments around the world, including the U.S., Sweden, Finland, and Australia, undertook large, randomized, controlled clinical trials (RCTs) in the 1960s and 1970s. Altogether, these trials tested the diet-heart hypothesis in about 67,000 people [6]. They typically tested levels of saturated fatty acids (SFA) considered to be standard at the time (between 12 and 18.3% of calories) versus lower amounts (7.7–11.2%), with replacement of SFA by polyunsaturated fatty acids (PUFA) from vegetable oils. Supplemental PUFAs were added to the experimental diets, such that the ratio of these two types of fatty acids (PUFA:SFA, known as the “P:S ratio”) differed greatly, from a low of 0.17 in the control group to a high of 2.44 in the experimental group [7] (p. 8). These trials were especially important, because, lasting between 1 and 7 years, they were considered to be of long enough duration to assess the impact on long-term clinical outcomes, i.e., “hard endpoints”, such as heart attacks and death. These outcomes are considered more reliable in making public health policy compared to studies that use “intermediary endpoints”, such as cholesterol or inflammatory measures, which are considered to be risk markers. Taken together, these core trials constitute the largest and longest experimental tests of the diet-heart hypothesis in the past 60 years since the hypothesis was introduced.

Remarkably, the diet-heart hypothesis gained widespread acceptance in the 1970s and 1980s, yet the results from the totality of these trials did not provide support for the hypothesis, as many critics at the time pointed out [8,9,10,11,12]. Perhaps for this reason, the clinical trial findings were largely ignored. A 2018 citation network analysis of RCTs found that “citation bias was common” from 1969 to 1984, with 82% of reviews supportive of the diet-heart hypothesis citing only the one clinical trial in favor of the hypothesis while ignoring three trials with contradictory results [13]. 

In 1977, the United States Senate Select Committee on Nutrition and Human Needs published the *Dietary goals for the United States,* widely known as the McGovern report [14]. Goal #5 of seven was to “reduce saturated fat consumption to account for about 10% of total energy intake …” This report led to establishment of the USDA-HHS policy, the Dietary Guidelines for Americans, first published in 1980 and every 5 years since. The first edition of the Guidelines issued seven recommendations, one of which advised Americans to “Avoid too much fat, saturated fat, and cholesterol” without recommending a specific numerical target. As mentioned, the 1990 and all subsequent DGAs have included a target for saturated fat as 10% of total calories or less. These 40 years of Guidelines have never incorporated the findings on saturated fat to emerge from the clinical trials conducted in the 1960s and 1970s, as discussed below.

The lack of a response to the data on saturated fat stands in contrast to the DGA’s revised position in response to two other bodies of scientific evidence to more accurately reflect the data. The 1980–2000 DGA recommendation [15] to consume less than 30% calories as fat was replaced in 2005 with the Institute of Medicine’s Recommended Dietary Allowance of 20 to 35% calories as fat [16] (p. viii). Similarly, the 300 mg cholesterol per day recommendation was abandoned in 2015, and although the 2020 DGA now states that healthy dietary patterns are “lower in cholesterol”, a systematic review by the USDA itself on the topic of dietary cholesterol specifically for the 2020 DGA concluded that there is “insufficient evidence” to link the intake of dietary cholesterol with cholesterol levels in the blood [17] (Part D, Chapter 9, p. 12). The 2020 DGA therefore contains conflicting messages, although the systematic review is more rigorous and therefore represents the more reliable conclusion.

### 2.1. Reviews of the Clinical Trial Data

According to a narrative review of the relevant literature, there have to date been at least 10 reviews of the RCTs addressing the effects of dietary saturated fat on CVD, with varying conclusions [6]. Those reviews that have found an unfavorable effect of saturated fats on CVD events included the Finnish Mental Hospital Study, in 4000 men and women, which showed a significant reduction in heart attacks and deaths among men on the experimental diet in one of the two hospitals included in the study, yet this effect was not seen in the other hospital, or for the women in the trial. This study also widely came to be recognized as not being randomized and was therefore excluded from most later reviews on saturated fats [6].

An updated systematic review by the Cochrane group in 2020 [18] on saturated fats reported that reducing dietary saturated fats reduced CVD events, but had no effect on the remaining seven CVD end-points including total mortality, CVD mortality, coronary heart disease (CHD) mortality, fatal heart attacks, non-fatal heart attacks, and CHD events. Even the significant effect of saturated fats on CVD events became nonsignificant when subjected to a sensitivity analysis that only included clinical trials which had successfully reduced saturated fat consumption while excluding those that intended to reduce saturated fat but were not successful. Thus, there were effectively no significant findings in the 2020 Cochrane review, which is consistent with an earlier Cochrane review on this topic, in 2015 [19]. Moreover, the collective RCT data did not find that these fats caused increased mortality [18]. Overall, therefore, there are serious concerns regarding the application of the RCT data for supporting the recommendation of a specific cap on dietary saturated fat intake.

### 2.2. Reviews of the Observational Data

Observational, or epidemiological, studies can demonstrate associations with disease outcomes, but may only be used to suggest cause-and-effect relationships when a number of criteria, such as consistency and strength of association, are met [20]. Observational data on the relation of saturated fat consumption with CHD have been collected since 1957, starting with the efforts of George Mann, from the University of Vanderbilt School of Medicine, who supervised the collection of dietary data from a 1049-person sample of the original Framingham Study [21,22]. Mann and colleagues reported that although men and women were consuming approximately 28% of total calories from animal fats, there was “no suggestion of any relation between diet and the subsequent development of CHD” [23]. These dietary results, comprising Section 24 of the Framingham study, were not published, an oversight that Mann later explained as part of a larger disregard for contradictory evidence in order to sustain a “diet/heart dogma” [24]. Subsequent collections of observational data over time now include information on nearly 350,000 individuals [25].

Reviews of this large body of evidence began in 2010 with a meta-analysis by Siri-Tarino et al., which concluded that available observational data provide “no significant evidence for concluding that dietary saturated fat is associated with an increased risk of CHD or CVD” [25]. There have since been at least eight meta-analyses of prospective observational studies on the relationship between saturated fat and heart disease, and these studies generally found no significant associations between consumption of these fats and CHD [6]. Most have excluded stroke as an endpoint in their analyses.

Two of the meta-analyses used statistical modeling (as opposed to experimental replacement in a randomized trial) to simulate replacement of SFAs with PUFAs and found an association with a lower risk of heart disease [26,27]. However, as noted above, observational studies in themselves are unable to demonstrate causal connections [20]. Moreover, they have significant limitations, including the potential for residual confounding or “healthy adherer bias”, the lack of available information on all potential confounders, measurement error in assessing habitual dietary consumption, social desirability bias, incomplete follow-up of participants, and publication biases [28]. A recent review of reviews, or “umbrella review”, of the observational data reported that replacing SFA with PUFA did “not convincingly reduce cardiovascular events or mortality” and that one “must consider that the diet-heart hypothesis is of uncertain validity” [29].

## 3. The Role of LDL-Cholesterol

Additional evidence used to support the continued recommendation for limiting saturated fat consumption comes from the well-demonstrated ability of SFAs to raise LDL-cholesterol concentration, which becomes evident when SFAs are replaced by PUFAs [30]. However, unlike LDL-cholesterol-lowering with drugs, cholesterol-lowering by diet has not reliably been shown to reduce cardiovascular outcomes [30]. The early core trials, for example, documented consistent reductions in total-cholesterol compared to minimal changes in the control groups, indicating a high level of compliance [7] (p. 9), yet even so, the evidence for benefit on longer-term clinical CVD outcomes is inconclusive [30].

Another explanation for why reductions in LDL-cholesterol concentration induced by saturated fat restriction have not been shown to reduce cardiovascular risk is that the decreased LDL-cholesterol primarily reflects reduced levels of large LDL particles [31]. As reviewed elsewhere [32], these particles have a weaker association with heart disease risk compared to small LDL particles which tend to be less affected by saturated fat restriction [31].

Moreover, saturated fat intake increases the levels of high-density lipoprotein (HDL) cholesterol, improving the ratio of total to HDL cholesterol, which is a robust marker of CVD risk [30]. Thus, due to the complex and multiple effects of saturated fats on blood lipids, the reliance on LDL-cholesterol alone as an indicator of SFA-mediated CVD risk is overly simplistic.

## 4. Current U.S. Dietary Guidelines for Saturated Fats

For the 2020 DGA process, the USDA excluded from consideration all systematic reviews conducted outside the agency, choosing instead to rely on novel reviews performed by the USDA staff. Thus, the approximately 20 review papers on saturated fats mentioned above which were published during the last decade by “external” scientists were excluded from consideration. Outside experts attempted to introduce this evidence via written comments to the USDA [33,34,35], pointing out the dramatic shift in thinking on saturated fats in the scientific community, a shift that implied the science on this topic can no longer be considered ‘settled.’ The 2020 DGAC briefly discussed these comments in one public meeting [36] (p. 37) but did not acknowledge the significant scientific disagreements on this topic in its final report.

By contrast, the 2015 DGAC had considered seven external review papers [37], yet there were some oversights in this 2015 review [38] For example, the 2015 DGAC excluded one paper with null findings on saturated fats [39] and instead relied heavily on a review paper [40] that included the Finnish Mental Hospital Study, which, as discussed earlier, was non-randomized and reported uniquely negative findings on SFAs and heart disease. The 2015 DGAC also included a review paper that looked exclusively at linoleic acid, not saturated fats [27]. Since the inception of the DGA in 1980, no DGAC has ever attempted its own, novel systematic review of the core trials from the 1960s and 1970s [38].

The most recent review, by the 2020 DGAC, concluded that there is “strong” evidence for continuing caps on saturated fat intake, and for replacing SFAs with PUFAs in particular. This conclusion relied on RCT data showing that replacement of SFAs with PUFAs could lower total and LDL-cholesterol concentrations [41] (pp. 16–17), without mentioning the limitation of relying solely on a surrogate measure such as LDL-cholesterol, as described above and despite extensive discussion of this issue in the scientific literature [31]. The DGAC also reported that increasing SFA in itself has a positive effect on HDL-cholesterol and that replacing it with PUFA has “predominantly null” effects on HDL cholesterol [41] (p.16). As noted above, this effect on HDL-cholesterol is an indicator that could offset any purported negative impact on CVD of the effect of SFA on LDL cholesterol.

For the findings on CVD events and CVD mortality as well as total mortality, the DGAC chose to rely exclusively on observational studies, despite the existence of more rigorous RCTs on this topic. As described above, observational studies cannot be used to establish a cause-and-effect relationship except upon fulfilling strict criteria, primarily when the strength of the association is both very strong and consistent. However, the overall strength of the associations found between SFAs and various cardiovascular endpoints and mortality have not been reported in the DGAC review and therefore cannot be evaluated. The consistency of findings is also uncertain.

For the DGAC’s primary conclusion, namely that saturated fats should be capped at 10% of calories, the USDA systematic review on the subject reviewed a total of 39 studies, all observational, on SFA (Table S1). Of these studies, 25 have null or negative findings, i.e., saturated fats were found either to have no effect on CVD or CHD endpoints, or their consumption was associated with a lower risk [41] (pp. 43–45). The DGAC also looked at eleven studies on SFA and stroke, eight of which had null findings and three of which found higher SFA intakes were linked to a lower risk of stroke. Thus, 88% of the findings did not support the DGAC conclusion on saturated fats and various heart disease outcomes. The same was found with the studies examining foods high in SFA. Sixteen studies, or 94% of the total, observed that dairy foods including butter either no had association or were negatively associated with CHD outcomes (i.e., higher dairy intake was associated with lower CHD risk). For meat, five studies showed no association and four showed a positive association. These analyses did not include the PURE study, because it did not meet the USDA inclusion criteria of “data only on countries ranked as high or very high in human development” [41] (p. 385). The PURE study, which observed 135,335 individuals from 18 countries in five continents [42], along with two systematic reviews of observational studies [43,44,45], one of which included 598,435 individuals, have all concluded that higher saturated fat consumption is not associated with increased risk of coronary heart disease, whereas it is associated with a lower risk of stroke.

The DGAC’s second recommendation on saturated fats, to replace SFA with PUFA, also appears to have relied exclusively on observational studies [41] (p. 385). The report’s accounting of these studies is somewhat confusing, but among them, we identified five studies based on mathematical modeling of presumed health effects, not direct observations of events, claiming that replacing SFA with PUFA was associated with lower CVD or CHD events. Seven studies, however, found either null results or that replacing PUFA with SFA was beneficial. The two replacement studies on stroke had either null findings or reported a higher risk of stroke mortality when PUFA was modeled to replace SFA. The majority of comparisons, therefore, did not find a conclusive benefit on heart disease of replacing SFA by PUFA.

Thus, the preponderance of evidence, as summarized here (Table S1), does not support the DGAC recommendation to limit saturated fat intake or to replace SFA with PUFA. Making a “strong” recommendation based on weak and contradictory evidence does not meet scientific standards for guidelines [45,46]. The conclusions based on the review of data on saturated fats also appear to be inconsistent with the statutory requirement by the U.S. Congress for the DGA to be based on “the preponderance of the scientific and medical knowledge which is current at the time the report is prepared” [3].

This statute also requires that the DGA be for the “general public”, yet the DGAC report notes that the studies on saturated fats reporting race or ethnicity were conducted mostly “in the U.S. or Scandinavia”, with “predominantly White or Caucasian participants” who were healthy middle-aged or older adults with overweight and without CVD at baseline [40] (pp. 41–42). This sample cannot be seen as representative of the racially and ethnically diverse population in the U.S., where nearly half are non-white and 60% of the population has been diagnosed with one or more chronic disease [47].

## 5. Importance of the Food and Diet Matrix

Saturated fats are increasingly being viewed as part of the food matrix and dietary patterns in which they appear naturally, rather than as an isolated nutrient [31,48]. Cheese and yogurt, for example, contain not only saturated fats but also other fatty acids, proteins, the milk fat globule membrane, potassium, and a number of essential nutrients including calcium, phosphorus, vitamins A, D, and B_12_, riboflavin, niacin and pantothenic acid. These nutrients interact with each other, and one can play a role in the effective absorption of another. For instance, fat-soluble vitamins such as A and D require fat for absorption. Moreover, the lack of consistent relationships of foods to CVD risk based on their saturated fat content is likely due in part to variation in effects of the overall food matrix and varying content of specific saturated fatty acids in these foods, as well as the dietary patterns in which they are consumed [31].

The matrix concept came to prominence in a 2010 international consensus statement [30]. Implementation of guidelines based on foods rather than nutrients has been promoted by the Food and Agricultural Organization (FAO), which has stated that today, “more than 100 countries” have developed or are currently developing “food-based guidelines” [49] These guidelines recognize that people consume foods, not nutrients, and furthermore, that they consume many of those foods in various amounts and combinations with each other.

In addition to the food matrix, the overall dietary pattern, particularly the level of carbohydrate, has an important impact on the way saturated fat is metabolized [31,50]. For example, if lowering saturated fat intake is achieved by consuming more carbohydrate, there is likely to be an adverse effect on CVD risk [51]. On the other hand, higher saturated fat intake in the context of a low-carbohydrate diet promotes less stimulation of insulin and greater oxidation of saturated fat. Such low-carbohydrate diets have been repeatedly shown to result in less accumulation of circulating saturated fatty acids [52,53] and improved diabetes [54] as well as cardiometabolic risk status [55].

The U.S. Dietary Guidelines have also moved away from nutrient-based recommendations, specifically with regard to total fat, yet the DGA has not done so for saturated fat. The Committee did, however, “recognize the importance of the growing body of research on specific fatty acids, food matrix and sources of fats, explicitly saturated fat” [17] (Part D, Chapter 9, p. 23).

## 6. Conclusions

Multiple reviews of the evidence have demonstrated that a recommendation to limit consumption of saturated fats to no more than 10% of total calories is not supported by rigorous scientific studies. Importantly, neither this guideline, nor that for replacing saturated fats with polyunsaturated fats, considers the central issue of the health effects of differing food sources of these fats. The 2020 DGAC review that recommends continuing these recommendations has, in our view, not met the standard of “the preponderance of the evidence” for this decision.

## Data Availability

The data used in this study are available online at: https://nesr.usda.gov/2020-dietary-guidelines-advisory-committee-systematic-reviews/dietary-fats-and-seafood-subcommittee/dietary-fat-cardiovascular-disease (accessed on 13 April 2021).

## Supplementary Materials

The following are available online at https://www.mdpi.com/article/10.3390/nu13103305/s1, Table S1: Findings from studies reviewed for 2020 US Dietary Guidelines on the associations between saturated fats and CVD/CHD events, stroke.
