# Supplementary material for: Dietary Saturated Fats and Health: Are the U.S. Guidelines Evidence-Based?
Source: Nutrients. 2021 Sep 22;13(10):3305. doi: 10.3390/nu13103305 (PMC8541481; doi:10.3390/nu13103305)
Supplement: Supplementary file 1 [file nutrients-13-03305-s001.zip › nutrients-1354130-supplementary.pdf]

Table S1: Findings from Studies Reviewed for 2020 US Dietary Guidelines on associations between saturated fats and CVD/CHD events, stroke

Studies with Direct Associations

	Total	Null result	Increased sat fats assoc. with lower CVD/CHD outcomes (negative result)	Null + Neg	Saturated fats associated with higher CVD/CHD outcome (positive result)	% support of Guidelines policy	% **do not** support Guidelines policy	
**Findings of associations between saturated fats and various endpoints**								
Total CVD Endpoints, incl. mortality	15	10	4	14	1	7%	93%	
CHD, CAD, MI, or PAD Endpoints	15	9	2	11	4	27%	73%	
Stroke	11	8	3	11	0	0%	100%	
**Total**	**41**	**27**	**9**	**36**	**5**	**12%**	**88%**	Total
**Findings of associations between foods and CVD/CHD endpoints (Stroke n/a)**								
Meat	9			5	4	**44%**	**56%**	Meat
CVD endpoints	2	1	0	1	1	50%	50%	
CHD etc endpoints	7	4	0	4	3	43%	57%	
Dairy	9			9	0	**0%**	**100%**	Dairy
CVD endpoints	2	1	1	**2**	**0**	0%	100%	
CHD etc endpoints	7	4	3	7	0	0%	100%	
Butter	**8**			**7**	**1**	**13%**	**88%**	Buter
CVD endpoints	3	3	0	3	0	0%	100%	
CHD etc endpoints	5	4	0	4	1	20%	80%	
Dairy + butter	**17**			**16**	**1**	**6%**	**94%**	

Modeling Studies

		Null result	Modeling of replacement associated with higher risk of outcome	Null + Neg	Modeling of replacement associated with lower risk of outcome	% support of Guidelines policy	% **do not** support Guidelines policy	
**Studies using modeling to approximate replacement effects**								
Replacing SFA with PUFA								
CVD Endpoints	6	2	1	3	3	50%	50%	
CHD, CAD, MI, or PAD Endpoints	6	3	1	4	2	33%	67%	
Total CVD and CHD	12			7	5	**42%**	**58%**	
Stroke mortality	2	1	1	2	0	0%	100%	
**Total**	**26**	**6**	**3**	**16**	**10**	**38%**	**62%**	Total

Notes:

Finding is only for women

Finding is for men only

Source: 2020 Dietary Guidelines Advisory Committee and Nutrition Evidence Systematic Review Team. Types of Dietary Fat and Cardiovascular Disease: A Systematic Review.
2020 Dietary Guidelines Advisory Committee Project. Alexandria, VA: U.S. Department of Agriculture, Food and Nutrition Service, Center for Nutrition Policy and Promotion, July 2020, pp. 16-17.
Available at: https://nesr.usda.gov/2020-dietary-guidelines-advisory-committee-systematic-reviews, pp. 43-45.
